# Frequencies of BCR::ABL1 Transcripts in Patients with Chronic Myeloid Leukemia: A Meta-Analysis

**DOI:** 10.3390/genes15020232

**Published:** 2024-02-12

**Authors:** Pablo Romero-Morelos, Ana Lilia González-Yebra, Daniela Muñoz-López, Elia Lara-Lona, Beatriz González-Yebra

**Affiliations:** 1 Department of Research, State University of the Valley of Ecatepec, Ecatepec 55210, Mexico State, Mexico; 2 Department of Applied Sciences to Work, Division of Health Sciences, University of Guanajuato, Campus León, León 37320, Guanajuato, Mexico; analilia@ugto.mx; 3 Department of Medicine and Nutrition, Division of Health Sciences, University of Guanajuato, Campus León, León 37320, Guanajuato, Mexico; daniela.lopez@ugto.mx (D.M.-L.); elia.lara@ugto.mx (E.L.-L.); 4 Research Unit, Bajío Regional High Specialty Hospital, León, Guanajuato, Blvd. Milenio, Col, San Carlos, León 37544, Guanajuato, Mexico

**Keywords:** chronic myeloid leukemia, BCR::ABL1 transcripts, *b3a2*, *b2a2*, *e1a2*, prevalence

## Abstract

Chronic myeloid leukemia (CML) is associated with the Philadelphia chromosome and distinct BCR::ABL1 gene transcripts. We assessed the frequencies of these transcripts in Mexico, Latin America, and worldwide. We determined the prevalence of BCR::ABL1 transcripts in CML patients and intercontinental or regional variations using specialized databases and keywords. We analyzed 34 studies from 20 countries, encompassing 5795 patients. Keyword-based searches in specialized databases guided data collection. ANOVA was employed for transcript distribution analysis. The *b3a2* transcript was most prevalent globally, followed by *b2a2*, with *e1a2* being the least frequent. Interestingly, Mexico City exhibited a higher incidence of *b2a2*, while *b3a2* predominated in the remaining country. Overall, no significant intercontinental or regional variations were observed. *b3a2* was the most common BCR::ABL1 transcript worldwide, with *b2a2* following closely; *e1a2* was infrequent. Notably, this trend remained consistent in Mexico. Evaluating transcript frequencies holds clinical relevance for CML management. Understanding the frequency of transcript informs personalized CML treatments.

## 1. Introduction

Leukemia has among the highest mortalities of any cancer, and chronic myeloid leukemia (CML) represents approximately 15–20% of all adult leukemias. CML is a hematologic neoplastic disease clinically characterized by a proliferation of mature myeloid cells and granulocytic precursors, both in the bone marrow and peripheral blood. The symptoms of CML are tiredness without reason, low body weight, pallor, and skin spots; however, most patients with CML do not present symptoms until the most advanced stages of this cancer. This disease is most often diagnosed in people over 60 years of age, with a higher percentage in men. There are three clinical stages for CML: the chronic phase (CML-CP), the accelerated phase (CML-AP), and the blast crisis (CML-BC). Without therapeutic intervention, the disease follows a natural progression from relatively benign CML-CP, through CML-AP, to terminal CML-BC. One essential feature of this neoplasia is the presence of the reciprocal translocation t(9;22)(q34;q11), which is found in approximately 90% of cases. This translocation gives rise to the Philadelphia chromosome (Ph), resulting in a head-to-tail fusion of the *BCR* and *ABL* genes. Consequently, a BCR::ABL1 chimeric gene is formed, encoding for a protein with an exacerbated tyrosine kinase activity. This gene fusion is a key point for CML pathogenesis because the oncoprotein activates signaling pathways such as RAS/MEK, JAK/STAT, and PI3K/AKT promoting cell growth, cell survival, and inhibition of apoptosis [1,2,3,4].

The BCR::ABL1 protein found in chronic myeloid leukemia (CML) incorporates various domains from both BCR and ABL1. Derived from BCR, these domains consist of an N-terminal coiled-coil domain (CC; amino acids 1–63), a Ser/Thr kinase domain containing a phosphorylated tyrosine 177 (Y177) docking site for the adaptor protein growth factor receptor-bound protein 2 (GRB2), and a Rho guanine nucleotide exchange factor (Rho/GEF) kinase domain (amino acids 298–413). On the other hand, domains from ABL1 include src homology (SH) domains (SH1/SH2), a proline-rich domain, and DNA- and actin-binding domains. Although distinct transcripts encode diverse proteins, a shared characteristic among all hybrid proteins is their constitutively active protein kinase activity in comparison to the wild-type ABL1 [5].

The most frequent breakpoint locations in the BCR gene are found in the major breakpoint cluster region (M-bcr), a genomic region of approximately 300 kb located between exons b2 and b3 or b3 and b4. Meanwhile, the breakpoint in the ABL gene is located in the 5’-region of exon a2. Depending on the rearrangement, both genes give rise to a combination of fusion messenger RNAs; for example, transcript *b2a2* (*e14a2*) is expressed when exon b2 is fused to exon a2, while transcript *b3a2* (*e13a2*) is expressed when exon b3 is fused with exon a2; however, other transcripts such as *e1a2* (exon e1 fused to exon a2) have been sporadically reported (approximately 1% of all CML) and show an inferior outcome to treatment with tyrosine kinase inhibitors (TKIs). These fusion transcripts have been proposed as central factors in CML pathogenesis and prognostic markers. However, their role remains unclear, as some authors have suggested that the *b3a2* rearrangement is associated with a shorter chronic phase and reduced survival, whereas conflicting reports have challenged this assertion. In this sense, Kjaer et al. and Bernardi et al. recently discussed and attributed this phenomenon to a technical issue, suggesting that differences between the expression of transcripts could be due to a technical limitation of RT-qPCR and not to biological causality, suggesting the use of digital PCR (dPCR) for better diagnosis and treatment of patients with chronic myeloid leukemia [4,6,7,8,9,10,11,12]. 

Furthermore, reports on the frequencies of these rearrangements are limited, although *b3a2* has been found in approximately 65% of CML cases, and *b2a2* accounts for only 35% of cases. However, cytogenetic studies in Mexico are limited and have focused only on the detection of the Ph chromosome [13,14,15,16,17,18,19]. Therefore, this meta-analysis aimed to determine the frequencies of BCR::ABL1 transcripts in patients diagnosed with CML in Mexico, Latin America, and worldwide and intercontinental or regional variations in these frequencies.

## 2. Materials and Methods

In this study, we used the PubMed and SciELO databases for conducting searches. The following keywords were employed in both Spanish and English: ‘LMC/CML’, ‘Leucemia Mieloide Crónica/Chronic Myeloid Leukemia’, ‘Rearreglo de transcritos/Transcript rearrangement’, ‘*b3a2*′, ‘*b2a2*′, ‘Citogenética/Cytogenetics’, ‘Cromosoma Filadelfia/Philadelphia Chromosome’, and ‘BCR::ABL1′. Only original articles published from 1997 to 2021, in either English or Spanish, were selected. These articles needed to include a search for at least *b3a2* and *b2a2* transcripts, identify the transcripts via reverse transcription polymerase chain reaction (RT-PCR), provide absolute or relative frequencies of transcript rearrangements, and involve a minimum of 10 samples.

Articles meeting the selection criteria were categorized according to the groups ‘Continents’ or the subgroups ‘Countries of the world’, ‘Latin America’, and ‘Mexico’. The subgroup ‘Mexico’ was further enriched with data from the Bajío Regional High Specialty Hospital (Hospital Regional de Alta Especialidad del Bajío) (HRAEB), including clinical data from the medical records of patients with Ph-positive CML and BCR::ABL1 rearrangement genotyping who were treated from 2007 to 2018.

### Data Analysis

Absolute frequencies for the b3a2, b2a2, and e1a2 transcripts were computed for countries covered in the chosen studies and various regions examined (continents, countries of the world, Latin America, and Mexico). Two-way analysis of variance (ANOVA) with Tukey’s multiple comparison test was conducted, employing a significance threshold of *p* < 0.05 and 95% confidence intervals. The objective was to identify significant variations in transcript distributions within the same country and across different countries. Statistical analyses were executed utilizing GraphPad Prism v.9.0.0, ensuring a robust and reliable examination of transcript frequency differences in the specified regions and countries.

## 3. Results

**Figure 1:**
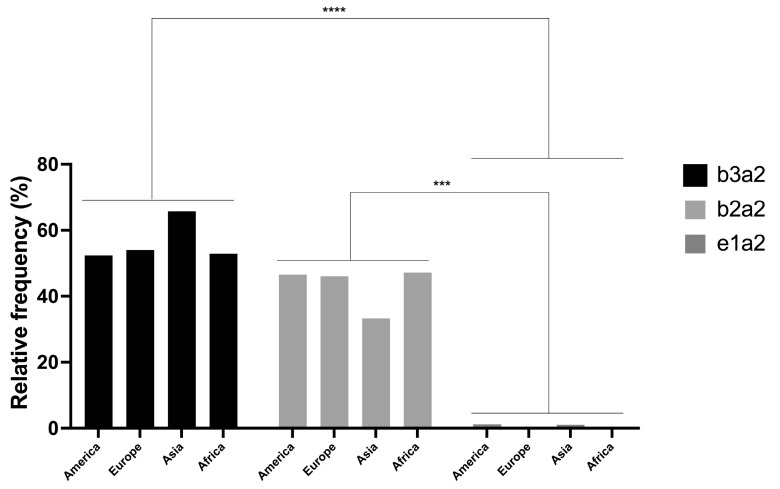
Distribution of frequencies of *b3a2*, *b2a2*, and *e1a2* transcripts for the analyzed continents. A two-factor ANOVA was conducted, with rows representing continents and columns representing transcripts. For the row-wise ANOVA, no significant differences were found (*p* > 0.99), while for the column-wise ANOVA, a *p*-value of < 0.0001 was obtained, indicating differences between the transcripts. Significant differences were observed only between the *b3a2* and *e1a2* transcripts (*p* < 0.0001) and between the *b2a2* and *e1a2* transcripts (*p* = 0.0002). *** *p* < 0.001; **** *p* < 0.0001.

This study included a total of 34 studies from 20 countries involving 5795 patients. According to the analysis of ‘Continents’, America contributed 9 studies (*n* = 1445 patients), Europe had 8 studies (*n* = 1445 patients), Asia had 15 studies (*n* = 2818 patients), and Africa had 2 studies (*n* = 87 patients). The Asian continent had the highest number of studies published up to the date of this study. The two-way ANOVA with Tukey’s multiple comparison test was applied to continents (rows) and transcript type (columns) to determine the presence of significant differences, showing significant differences between the *b3a2* and *e1a2* transcripts and between the *b2a2* and *e1a2* transcripts (Table 1). Moreover, the *e1a2* transcript had the lowest frequency in all continents (mean frequency: 0.55%), whereas the *b3a2* and *b2a2* transcripts occurred in statistically significantly similar proportions, although *b3a2* (mean frequency: 56.21%) was clearly the most frequent transcript in all the continents analyzed (Figure 1).

The subgroup ‘Countries of the world’ consisted of Mexico with five studies, including data from the HRAEB (*n* = 646); Pakistan (*n* = 135) and Italy (*n* = 222), with three studies each; Brazil (*n* = 259), Iran (*n* = 142), Korea (*n* = 652), Malaysia (*n* = 76), India (*n* = 1395), and Germany (*n* = 1058), with two studies each; and USA (*n* = 396), Syria (*n* = 15), Indonesia (*n* = 177), Canada (*n* = 144), England (*n* = 71), Iraq (*n* = 98), France (*n* = 32), Sudan (*n* = 43), Tunisia (*n* = 44), Saudi Arabia (*n* = 128), and Poland (*n* = 62), with one study each.

Regarding the frequencies of different BCR::ABL1 transcripts in the included countries, the *b3a2* variant was the most prevalent in most of the cases included in this study (*n* = 3433 patients), followed by *b2a2* (*n* = 2316 patients) and *e1a2* (*n* = 46 patients). However, *b2a2* was the most prevalent variant in some Middle Eastern countries, such as Syria and Sudan, with frequencies of 80% (*n* = 12) and 58% (*n* = 25), respectively, followed by the *b3a2* variant (Table 2).

**Figure 2:**
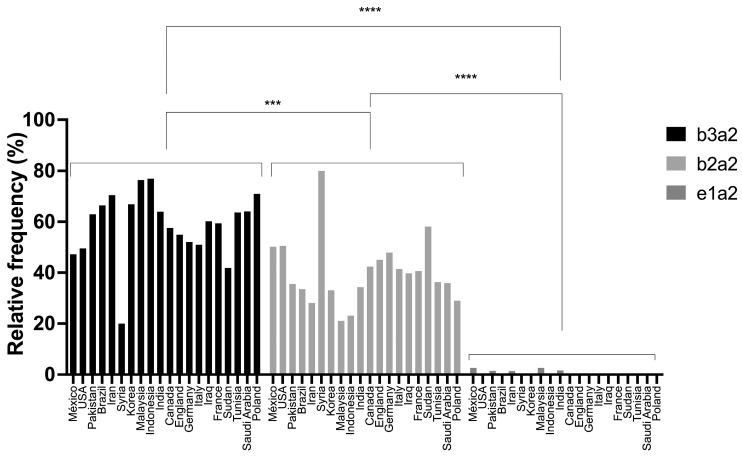
Histogram of frequencies of the different BCR::ABL1 transcripts in countries of the world. A two-factor ANOVA was conducted, with rows representing countries and columns representing transcripts. For the country-wise ANOVA, no significant differences were found (*p* > 0.999), while for the transcript-wise ANOVA, a *p*-value of <0.0001 was obtained. Significant differences were observed in all countries between the *b3a2* and *e1a2* transcripts (*p* < 0.0001) and between *b3a2* and *b2a2* transcripts (*p* = 0.0002), with *b3a2* being the most frequent transcript. Significant differences were also found for the *b2a2* transcript when compared to the *e1a2* transcript (*p* = 0.0001). *** *p* < 0.001; **** *p* < 0.0001.

The two-way ANOVA with Tukey’s multiple comparison was applied to determine the presence of significant differences in the frequencies of the transcripts in the countries of the world, showing with statistical significance that *e1a2* had the lowest frequency and *b3a2* had the highest frequency, although *b2a2* only showed significant differences when compared with *e1a2* (Figure 2). Notably, in the cases of Syria and Sudan, *b2a2* seemed to be the most common variant, but there was not enough significant evidence to confirm this claim, especially considering that the Syrian study included only 15 patients and the Sudanese study included only 43.

In the case of Latin America, a total of 14 reports were included, involving 1386 patients with CML: two studies from Cuba (*n* = 271), one from Venezuela (*n* = 81), one from Brazil (*n* = 21), one from Bolivia (*n* = 250), one from Chile (*n* = 19), one from Guatemala (*n* = 34), one from Ecuador (*n* = 37), one from Colombia (*n* = 27), and five from Mexico, including data from the HRAEB (*n* = 646).

The analysis of transcript frequencies showed that *b3a2* was the most frequent variant in most Latin American countries included in this study, with relative frequencies ranging from 44% to 73%. However, in Colombia and Ecuador, the frequencies were 37% (*n* = 10) and 5.4% (*n* = 2), respectively, with *b2a2* emerging as the most frequent variant in these two countries. Interestingly, the *e1a2* transcript was the least frequent, with a frequency of <4% in most Latin American countries. However, Guatemala and Chile had frequencies of 11% (*n* = 4) and 26% (*n* = 5), respectively (Table 3).

Subsequently, to determine significant differences in the distribution of different transcripts among Latin American countries, a two-factor ANOVA was conducted, with countries as rows and transcript types as columns. Interestingly, significant differences were observed only between different transcripts (*p* = 0.0014). However, Tukey’s multiple comparison showed no significant differences (*p* > 0.99) between the frequencies of each transcript in the countries analyzed, despite the clear differences in the frequencies of countries such as Colombia, Ecuador, and Chile. Nevertheless, the prevalence of *b2a2* in Ecuador was approximately 95%, suggesting the need for further studies to confirm this distribution, especially given that the analyzed study included only 37 patients.

**Figure 3:**
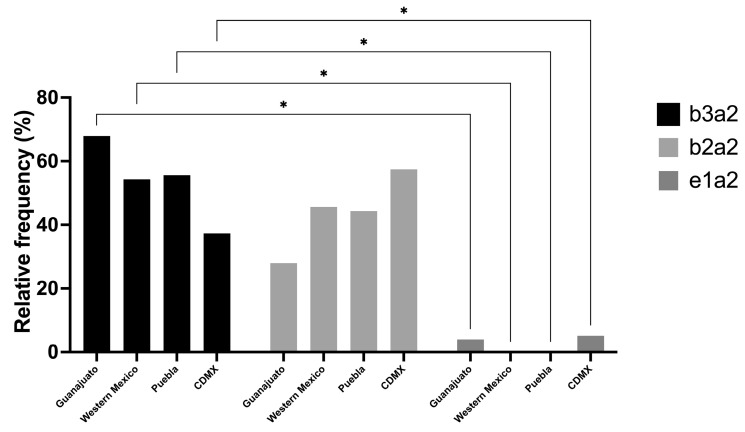
Histogram of frequencies of the different BCR::ABL1 transcripts in different regions of Mexico. A two-factor ANOVA was conducted, with rows representing regions and columns representing transcripts. For the regional ANOVA, no significant differences were found (*p* > 0.099), while for the transcript-wise ANOVA, a *p*-value of 0.0025 was obtained, indicating significant differences in the Tukey multiple comparison for the frequency between *b3a2* and *e1a2* transcripts. In the case of Guanajuato, the western region of Mexico, and Puebla, the *b3a2* transcript was significantly more frequent compared to *e1a2* (0.0202). In Mexico City (CDMX), the most frequent transcript was *b2a2*. Meanwhile, no significant differences were noted between the *b3a2* and *b2a2* transcripts for any of the regions analyzed. CDMX = Mexico City. * *p* < 0.05.

Finally, to determine the frequency of different transcripts in Mexico, data from the five included reports were examined, and the regions they originated from were determined, with two studies from Mexico City (*n* = 308); one study from Puebla (*n* = 232); one study from western Mexico, with patients from Jalisco, Colima, Michoacan, and Nayarit (*n* = 81); and data from the HRAEB in Guanajuato (*n* = 25). The two-way ANOVA test was applied to determine the presence of significant differences in the frequencies of the transcripts (columns) depending on the region (rows), but no significant differences were found between regions (*p* > 0.999). However, the Tukey’s multiple comparison test showed significant differences between transcript variants (*p* = 0.0025), with *b3a2* (*p* = 0.02) having the highest frequency in the regions of Guanajuato, western Mexico, and Puebla, compared with *e1a2*. However, no significant differences were found between the *b2a2* and *b3a2* transcripts. Notably, Mexico City displayed a slightly different pattern compared to the findings in the other regions analyzed; *b2a2* was the most frequent transcript with 57.5% of the cases (*n* = 177), followed by *b3a2* with 37.3% of the cases (*n* = 115) (Figure 3).

## 4. Discussion

At a global level, cancer remains a significant public health problem, positioning itself as one of the leading causes of death. However, advances in the diagnosis and treatment of these types of diseases have contributed to a drastic reduction not only in the number of cases but also in the annual death toll. This reduction can be attributed to factors such as increased access to healthcare services, early diagnosis, improvements in the monitoring of neoplastic diseases, and better management.

Nevertheless, one of the primary challenges in understanding cancer lies in population differences, impacting both treatment and diagnostic criteria. These differences stem from ethnic, genetic, socioeconomic, and even environmental factors. Therefore, exploring genetic variations at the population level becomes extremely important for the comprehension and improved management of cancer diseases.

Several diseases are caused by gene variants and show epidemiological differences depending on geographic location or ethnicity, but similarities between ethnic groups have been demonstrated in some cases in different regions of the world. In this sense, our work found some similarities in the frequency of BCR::ABL1 transcripts in CML patients, in the special case between Syria and Sudan, showing a high frequency of the *b2a2* transcript, which may be caused by the migration of the Sudanese population to Syria due to armed conflicts in the region. This has resulted in a high number of Sudanese refugees in Syria. However, this premise needs to be evaluated, considering current displacements in the Middle East regions. 

On the contrary, in the Americas, the distribution of transcript variants among Latin American patients with chronic myeloid leukemia (CML) was generally uniform, showing no notable distinctions. Nevertheless, certain countries examined in this research exhibited skewed outcomes that might imply substantial variations. Notably, in Chile and Ecuador, there were notable deviations, with higher relative frequencies observed for b3a2 (73.7%) and b2a2 (94.6%) transcripts, respectively. These findings suggest the presence of potential differences that warrant more precise investigations to validate this phenomenon. Therefore, more detailed studies are essential to ascertain and confirm the observed disparities, emphasizing the need for a thorough examination of transcript variant distributions in different regions of Latin America [47,56,57].

With the exception of the findings in Chile and Ecuador, it can be suggested that, as expected due to the ethnic origins of Latin American populations, similar transcript distributions may be encountered. Studies on the interethnic admixture and evolution of Latin American populations have shown similar ancestries. For example, all Latin Americans share a significant proportion of Native American ancestry, reflecting the region’s indigenous heritage and emphasizing that the first inhabitants of the Americas were Native Americans who migrated from Asia across the Bering land bridge. In addition, many Latin Americans also have European ancestry, reflecting the legacy of European colonization. These similarities are likely due to the shared history of the region. All Latin American populations have been shaped by the same forces of migration, colonization, and intermarriage. As a result, they share a common genetic heritage that reflects the region’s rich and complex history. Regarding this, countries such as Cuba, Venezuela, and Brazil have mainly African and European genetic contributions and showed similar distributions of the transcript variants in this study. Countries such as Mexico and Colombia which mainly have European and Amerindian genetic contributions also showed similar distributions of the transcripts. Although the Latin American colonization processes were complex, the ethnic similarity throughout Latin America is notorious. As a result, the distributions of transcript variants are also very similar, except for that of Ecuador, which has a higher Amerindian ancestry, and Chile, which has a higher European ancestry [56,58,59].

Likewise, in the examination of ancestry across various regions of Mexico, distinct variations were identified. Mexico exhibits predominant genetic contributions from European and Amerindian ancestry, albeit in varying ratios depending on the state. Notably, Mexico City displays nearly equal proportions of Amerindian and European ancestry, whereas Puebla and the western states of Mexico primarily feature Amerindian ancestry. This discrepancy in ancestry distribution may elucidate the minor distinctions observed between these regions [60,61,62].

Racial or ethnic similarities regarding the distribution of BCR::ABL1 transcripts in CML can be masked in certain circumstances, such as the current development of mobility technologies and human settlement due to high world migration rates, a consequence of globalization. In this respect, hereditary diseases behave quite differently from infectious diseases, especially regarding epidemiological changes attributed to migration since changes in the geographical distribution of hereditary diseases occur over long periods. Thus, one must be cautious when determining the dominant BCR::ABL1 transcript variants and the prognosis of the disease based solely on the ethnic history of a country or region [63,64,65,66]. Additionally, it is important to also consider environmental factors, as, at least in this study, they were not an explored variable; however, they could be crucial for understanding the differences and similarities among different transcript types present in populations. Therefore, it is also suggested to conduct studies that incorporate the environmental status and the presence of pollution markers that may be related to the preferential expression of a particular BCR::ABL1 transcript.

The data obtained in this study suggest that the *b3a2* variant is the most frequent BCR::ABL1 gene transcript in patients with CML in the worldwide population, followed by *b2a2* and *e1a2*. Variations in this order exist in some countries around the world and in the Latin American continent itself. These could be partially explained by ethnic differences, as mentioned previously, but further in-depth studies should be conducted to better understand these aspects of CML.

The presence of BCR::ABL1 transcript variants in CML has implications for the disease in terms of diagnosis, prognosis, and patient survival [4,6,7,8,9,10]. Some studies have reported that the *b3a2* variant is associated with worse prognosis and survival compared with *b2a2*. Although there is contradictory literature regarding the association of transcript types in the prognosis, treatment, and survival of patients with CML, it is important to be aware of their distribution worldwide and in our country. Some reports have suggested different tyrosine kinase inhibitor (TKI) treatment regimens based on the expressed transcripts, although these claims still need to be validated with more in-depth studies. However, it is important to mention that the presence of the *e1a2* transcript has been linked to diagnoses at older ages and an increased risk of progression to the blast phase, thus leading to a poorer response to TKI treatments [67].

## 5. Conclusions

Determining the frequencies of BCR::ABL1 transcripts in CML has implications for CML diagnosis, and potentially for prognosis and patient survival. The most frequent transcript worldwide was *b3a2*, followed by *b2a2* and *e1a2*. The frequency of *e1a2* is higher in some Asian countries compared with that in the rest of the world. In Mexico, there are no significant differences in this trend. Further studies are warranted to determine the role played by ethnicity in the presence of BCR::ABL1 gene variants, the role of these variants in the course of the disease, and the clinical impact of these variants.

## Figures and Tables

**Table 1:** Tukey’s multiple comparison results for BCR::ABL1 transcript frequencies in the different continents based on 34 reports [6,7,10,13,20,21,22,23,24,25,26,27,28,29,30,31,32,33,34,35,36,37,38,39,40,41,42,43,44,45,46,47,48,49].

			Mean Difference	95% CI	*p*-Value
		*b2a2*			
*b3a2*	America	America	14.85	−5.472–35.17	0.1752
	America	Europe		−16.19–45.89	0.5501
	America	Asia		−16.19–45.90	0.5499
	America	Africa		−16.19–45.89	0.5501
	Europe	Europe		−5.472–35.17	0.1752
	Europe	Asia		−16.19–45.90	0.5499
	Europe	Africa		−16.19–45.89	0.5501
	Asia	Asia		−5.472–35.17	0.1752
	Asia	Africa		−16.20–45.89	0.5504
	Africa	Africa		−5.472–35.17	0.1752
			Mean difference	95% CI	*p*-value
		*e1a2*			
*b2a2*	America	America	41.96	21.64–62.28	0.0012 *
	America	Europe		10.92–73.00	0.0118 *
	America	Asia	41.97	10.92–73.01	
	America	Africa	41.96	10.92–73.00	
	Europe	Europe		21.64–62.28	0.0012 *
	Europe	Asia	41.97	10.92–73.01	0.0118 *
	Europe	Africa	41.96	10.92–73.00	
	Asia	Asia		21.64–62.28	0.0012 *
	Asia	Africa		10.92–73.00	0.0118 *
	Africa	Africa		21.64–62.28	0.0012 *
			Mean difference	95% CI	*p*-value
		*e1a2*			
*b3a2*	America	America	56.81	36.49–77.13	0.0002 *
	America	Europe		25.77–87.85	0.0024 *
	America	Asia	56.82	25.77–87.86	
	America	Africa	56.81	25.77–87.85	
	Europe	Europe		36.49–77.13	0.0002 *
	Europe	Asia	56.82	25.77–87.86	0.0024 *
	Europe	Africa	56.81	25.77–87.85	
	Asia	Asia		36.49–77.13	0.0002 *
	Asia	Africa		25.77–87.85	0.0024 *
	Africa	Africa		36.49–77.13	0.0002 *

* Significant values *p* < 0.05.

**Table 2:** Relative frequency of BCR::ABL1 transcripts in countries of the world based on 34 reports [6,7,10,13,20,21,22,23,24,25,26,27,28,29,30,31,32,33,34,35,36,37,38,39,40,41,42,43,44,45,46,47,48,49].

	*n*	*b3a2* % (*n*)	*b2a2* % (*n*)	*e1a2* % (*n*)
Mexico	646	47.21 (305)	50.15 (324)	2.63 (17)
USA	396	49.49 (196)	50.51 (200)	0.00 (0)
Pakistan	135	62.96 (85)	35.56 (48)	1.48 (2)
Brazil	259	66.41 (172)	33.59 (87)	0.00 (0)
Iran	142	70.42 (100)	28.17 (40)	1.41 (2)
Syria	15	20.00 (3)	80.00 (12)	0.00 (0)
Korea	652	66.87 (436)	33.13 (216)	0.00 (0)
Malaysia	76	76.32 (58)	21.05 (16)	2.63 (2)
Indonesia	177	76.84 (136)	23.16 (41)	0.00 (0)
India	1395	63.94 (892)	34.41 (480)	1.65 (23)
Canada	144	57.64 (83)	42.36 (61)	0.00 (0)
England	71	54.93 (39)	45.07 (32)	0.00 (0)
Germany	1058	52.08 (551)	47.92 (507)	0.00 (0)
Italy	222	57.21 (127)	42.79 (95)	0.00 (0)
Iraq	98	60.20 (59)	39.80 (39)	0.00 (0)
France	32	59.38 (19)	40.63 (13)	0.00 (0)
Sudan	43	41.86 (18)	58.14 (25)	0.00 (0)
Tunisia	44	63.64 (28)	36.36 (16)	0.00 (0)
Saudi Arabia	128	64.06 (82)	35.94 (46)	0.00 (0)
Poland	62	70.97 (44)	29.03 (18)	0.00 (0)
N	5795	59.24 (3433)	39.97 (2316)	0.79 (46)

**Table 3:** Relative frequencies of BCR::ABL1 transcripts in Latin American countries based on 14 reports [14,15,44,45,46,47,50,51,52,53,54,55].

	*n*	*b3a2* % (*n*)	*b2a2* % (*n*)	*e1a2* % (*n*)
Mexico	646	47.2 (305)	50.2 (324)	2.6 (17)
Cuba	271	56.5 (153)	41.0 (111)	2.6 (7)
Venezuela	81	55.6 (45)	44.4 (36)	0.0 (0)
Brazil	21	57.1 (12)	42.9 (9)	0.0 (0)
Bolivia	250	61.6 (154)	38.4 (96)	0.0 (0)
Chile	19	73.7 (14)	0.0 (0)	26.3 (5)
Guatemala	34	44.1 (15)	44.1 (15)	11.8 (4)
Colombia	27	37.0 (10)	59.3 (16)	3.7 (1)
Ecuador	37	5.4 (2)	94.6 (35)	0.0 (0)
N	1386	51.2 (710)	46.3 (642)	2.5 (34)

## Data Availability

The data that support the findings of this study are available on request from the corresponding author. The data are not publicly available due to the data also forms part of an ongoing study.
